# Acceptance of Mobile Health Applications: Examining Key Determinants and Moderators

**DOI:** 10.3389/fpsyg.2019.02791

**Published:** 2019-12-10

**Authors:** Andreia Nunes, Teresa Limpo, São Luís Castro

**Affiliations:** Center for Psychology at University of Porto, Faculty of Psychology and Education Sciences of the University of Porto, Porto, Portugal

**Keywords:** technology acceptance, mobile health applications, UTAUT model, smartphone, human-technology interaction

## Abstract

Mobile health applications are increasingly numerous and varied. However, despite high expectations and large budgets involved in their development they are often rejected by potential users, and little is known on why this happens. This study aimed to fill this gap by investigating the determinants of technology acceptance and its moderators. Aligned with the Unified Theory of Acceptance and Use of Technology, we examined the moderating roles of age, gender, and smartphone experience in the relationship between technology acceptance determinants (performance expectancy, effort expectancy, social influence, and facilitating conditions) and the intention to use mobile health applications (*N* = 394, 18–65 years). A stepwise multiple linear regression was conducted. Results showed that the intention to use mobile health applications was determined by performance expectancy moderated by age and smartphone experience, and that the role of the other determinants depended on age and gender (e.g., more intention to use in older men if less effort, and in younger men if better facilitating conditions). These findings show that user characteristics are relevant moderators and should be considered when targeting specific populations to use mobile health applications.

## Introduction

Recent progress on Information and Communication Technologies (ICT), specially on mobile devices, has pervasively influenced our daily life (Huang and Kao, 2015). The smartphone is now routinely used in such diverse areas as work, education, entertainment, communication, and healthcare (Verkasalo et al., 2010; Ma et al., 2016). The fast-paced development and ubiquity of ICT contrasts with the rather slow-paced scientific validation of its products, as research into ICT can barely keep up with digital industry and user demands (Boudreaux et al., 2014). This is particularly evident in the development of mobile applications (hereafter, apps) in the health domain, commonly referred to as mHealth. The number of mobile health apps has notoriously increased in the past years, with no less than 325,000 mHealth apps available in 2017 (information retrieved from Research2Guidance, 2017)^1^.

The main goal of mHealth apps is to improve health outcomes through active self-management and involvement in healthcare. These apps assist users in monitoring overall wellness and preventing and/or managing disease (Aitken, 2015). However, despite the potential benefits of mHealth apps, few studies have supported their effectiveness (Byambasuren et al., 2018). Indeed, various studies reveal many of these apps have narrow functionalities, tend to provide information to users rather than engage them behaviorally, are not always suitable to the target public, and were tested with small sample sizes for a short period of time (Aitken, 2015; Byambasuren et al., 2018).

Given this state of affairs, it is important to identify key factors influencing the acceptance of mHealth apps so that software developers can fine-tune their design and improve their relevance and user friendliness. The present study aims to examine the association of ICT acceptance determinants with the behavioral intention to use mHealth apps, and to clarify potential moderators of this relationship.

A variety of theoretical models have been developed to explain the factors that influence acceptance and use of ICT (Davis, 1989; Venkatesh et al., 2003, 2012). Venkatesh and colleagues proposed the Unified Theory of Acceptance and Use of Technology model (Venkatesh and Davis, 2000; Venkatesh et al., 2003; Venkatesh and Bala, 2008). This model has been tested in education, organization and health settings (Huang and Kao, 2015; Williams et al., 2015; Venkatesh et al., 2016); it is widely accepted as a valuable framework for understanding ICT acceptance that also holds in different cultures (American and Asian; Dwivedi et al., 2016). The UTAUT model assumes that behavioral intentions and effective use of technology are influenced by four acceptance determinants: performance expectancy, effort expectancy, social influence, and facilitating conditions (Venkatesh, 2000; Venkatesh et al., 2003, 2016). In a recent review of the literature on UTAUT from 2003 to 2014, Venkatesh et al. (2016) conclude that this model has been of special importance because it withstood rigorous empirical validation and impelled further theoretical development in technology acceptance and use.

Performance expectancy refers to the person’s beliefs about the benefits of using ICT (Venkatesh et al., 2003, 2012). This determinant has been identified as the strongest predictor of the behavioral intention to use ICT in several settings (Venkatesh et al., 2003; Kijsanayotin et al., 2009; Cimperman et al., 2016; Hsu and Wu, 2017). Several studies have shown that the more a mHealth app is perceived as bringing benefits to one’s health, the stronger the intention to use it (Carlsson, 2006; Sun et al., 2013; Hoque and Sorwar, 2017).

Effort expectancy refers to the extent to which a particular ICT is perceived as easy to use (Venkatesh et al., 2003, 2012). Effort expectancy is a known predictor of the intention to use ICT in consumer contexts when the user has little or no experience with the ICT in question (Venkatesh et al., 2012). In health-related areas, it has also been shown that the intention to use is greater when mHealth apps have low effort expectancy (Sun et al., 2013).

Social influence is defined as the extent to which an individual believes that important others would support him/her in using ICT (Venkatesh et al., 2003, 2012). This determinant was found to be positively associated with the intention to use digital information and mobile health services in healthcare (Wills et al., 2008; Sun et al., 2013).

Facilitating conditions focuses on the belief that technical support will be available to perform a behavior required by the ICT (Venkatesh et al., 2003, 2012). This determinant is a significant predictor of the intention to use ICT: if individuals believe there is a trustworthy support system to assist them, their intention to use mHealth apps is greater than when such a perceived support is lacking (Yi et al., 2006; Bhattacherjee and Hikmet, 2008).

In spite of the importance of the UTAUT, other models aimed to explain the factors that influence individuals’ acceptance and use of ICT have been proposed: Technology Acceptance Model (TAM; Davis, 1989; Venkatesh and Davis, 1996), the TAM 2 (Venkatesh and Davis, 2000), the TAM 3 (Venkatesh, 2000; Venkatesh and Bala, 2008), and the UTAUT 2 (Venkatesh et al., 2012). These models included ICT determinants not present in the UTAUT model (privacy, emotion, and previous exposure to technology), which have also been explored in qualitative research (Psychoula et al., 2018). Privacy is an important factor in health-related technologies, such as smart homes (Singh et al., 2018) and person tracking devices (Holzinger et al., 2008). Emotion was found to be related with ICT performance in studies about emotion detection in usability testing software (Stickel et al., 2009). This emotional aspect of ICT has already been examined under the TAM 3, through the construct of computer anxiety (Venkatesh, 2000). Previous exposure to technology seemed positively related with the enjoyment people have with technology (Holzinger et al., 2010).

The effects of these determinants can be either amplified or constrained by moderators such as age, gender, experience and voluntariness^2^ (Venkatesh et al., 2003; Arenas-Gaitán et al., 2015). Despite the robustness of the determinants included in the UTAUT model, research has barely considered these moderating effects (Venkatesh et al., 2016).

Age was proposed to have a key moderating effect on the behavioral intention to use ICT (Venkatesh et al., 2003, 2012; Magsamen-Conrad et al., 2015). Despite being the main predictor across all ages, the effects of performance expectancy seem to be stronger for younger rather than older adults (Wang and Wang, 2010; Venkatesh et al., 2012; Cimperman et al., 2016). An opposite pattern was found for the other determinants. For adults aged more than 50 years it was effort expectancy, social influence and facilitating conditions that stood out as factors influencing the intention to use ICT (Venkatesh and Morris, 2000; Venkatesh et al., 2003; Cimperman et al., 2016).

Gender has been widely studied as a main demographic variable related to ICT adoption (Parameswaran et al., 2015). Venkatesh et al. (2003) suggested that performance expectancy is the strongest predictor for men, whereas effort expectancy, social influence, and facilitating conditions are the strongest predictors for women.

Experience refers to the previous experience that a person has had with the ICT that is being studied (Parameswaran et al., 2015). As age and gender, experience also seems to affect the four determinants of ICT acceptance (Igbaria et al., 2015). For example, effort expectancy seems to depend upon prior ICT experience (Parameswaran et al., 2015). Moreover, Taylor and Todd (1995) suggested that people with less ICT experience may be more prone to be influenced by others and to give more value to technical assistance. Having less experience means less familiarity and knowledge about ICT, which may increase reliance on external support (Alba and Hutchinson, 1987).

As reviewed above, ICT acceptance can be influenced by a set of determinants and potential moderators. However, the role of these moderators has been neglected (Williams et al., 2015). Here, we aim to fill this gap by examining the determinants affecting ICT acceptance and its relationship with potential moderators. We expect that the effects of the four determinants on the behavioral intention to use mHealth apps would be moderated by age, gender, and smartphone experience.

## Materials and Methods

### Participants

There were 574 adult participants in this study. We set two exclusion criteria: not using a smartphone, or already using mHealth apps. Based on this, 171 participants were dropped from the initial subject pool. Nine participants were also excluded because they did not respond to at least one item of the questionnaire. The final sample included 394 participants aged between 18 and 65 years (*M* = 35.55, *SD* = 15.03; 74% women). About half were Psychology undergraduate or graduate students (49.5%). Of the remaining participants, 6% had completed primary education, 10% upper primary, 18% middle education, 29% secondary education, and 37% had a university degree. The study was approved by the Departmental Ethics Committee, and written informed consent was obtained from all participants.

### Materials and Procedure

We used a questionnaire with two parts: one with questions about age, gender, education, smartphone and mHealth apps experience, and another about ICT acceptance determinants.

To assess smartphone experience, participants were asked to indicate how often they used the following functions: making calls, writing text messages, playing games, being engaged in social networks, using Internet, sending e-mails, taking pictures, and using the agenda. Answers were given in a scale ranging from 1 (*never*) to 7 (*several times a day*). The final variable “smartphone experience” was computed by averaging the frequency of use of all these functions.

To assess the four determinants of ICT acceptance and the behavioral intention to use mHealth apps, we used the Questionnaire of Acceptance of Technology – mHealth Apps (Supplementary Table S1), adapted from a previously validated scale by Cimperman et al. (2016). Because this scale was directed at home telehealth devices, we rephrased the items to focus on mHealth apps. A brief definition of mHealth applications was provided at the beginning of the questionnaire (cf. Supplementary Table S1). The questionnaire is composed of 19 items grouped into five factors: performance expectancy (α = 0.88), effort expectancy (α = 0.76), social influence (α = 0.79), facilitating conditions (α = 0.69), and behavioral intention to use mHealth apps (α = 0.94). Participants indicated their level of agreement with the statements using a 7-point scale (1 = *strongly disagree*; 7 = *strongly agree*).

The questionnaire was initially administered to undergraduates in classroom groups of about 30 students, who were asked to take an additional questionnaire with them and administer it to a person aged between 40 and 65 years.

## Results

### Descriptive Statistics and Correlations

Table 1 presents means and standard deviations for all variables, together with bivariate correlations. Smartphone experience and effort expectancy correlated negatively with age and with gender. All ICT acceptance determinants were positively correlated with each other. Behavioral intention correlated positively with all variables except gender and smartphone experience.

**TABLE 1:** Means, standard deviations, and correlations for all variables.

	**Correlations**							
**Measures**	**1**	**2**	**3**	**4**	**5**	**6**	**7**	**8**
1. Age								
2. Gender^a^	0.25^∗∗∗^							
3. Smartphone experience	–0.45^∗∗∗^	–0.16^∗∗^						
4. Performance expectancy	0.09	–0.07	0.09					
5. Effort expectancy	–0.29^∗∗∗^	–0.18^∗∗∗^	0.32^∗∗∗^	0.51^∗∗∗^				
6. Social influence	–0.07	−0.13^∗^	0.05	0.57^∗∗∗^	0.53^∗∗∗^			
7. Facilitating conditions	–0.02	–0.07	0.06	0.41^∗∗∗^	0.61^∗∗∗^	0.57^∗∗∗^		
8. Behavioral intention	0.20^∗∗∗^	–0.06	0.05	0.82^∗∗∗^	0.43^∗∗∗^	0.50^∗∗∗^	0.39^∗∗∗^	
*M*	35.55	0.26	4.72	4.43	5.17	4.80	5.07	4.17
SD	15.03	0.44	1.17	1.01	0.84	0.94	0.80	1.23

*^*a*^Dummy coded, 0 = female, 1 = male. ^∗^*p* < 0.05, ^∗∗^*p* < 0.01, and ^∗∗∗^*p* < 0.001.*

### Prediction of Behavioral Intention to Use mHealth Apps

To examine the moderating roles of age, gender, and smartphone experience in the relationship between ICT acceptance determinants and the behavioral intention to use mHealth apps, we conducted a stepwise multiple linear regression (see Table 2 for the final model).

**TABLE 2:** Final model with all main effects and interactions of age, gender, smartphone experience, and ICT acceptance determinants on participants’ behavioral intention to use mHealth.

**Predictors**	***B***	**SE**	**β**	***t***
Age	0.01	0.003	0.18	4.39^∗∗∗^
Gender	–0.01	0.10	–0.002	–0.05
Smartphone experience	0.06	0.05	0.06	1.27
Performance expectancy	0.96	0.06	0.79	15.52^∗∗∗^
Effort expectancy	0.07	0.08	0.05	0.84
Social influence	0.04	0.07	0.03	0.48
Facilitating conditions	0.01	0.08	0.01	0.17
Age × gender	0.003	0.007	0.02	0.41
Age × smartphone experience	–0.002	0.003	–0.02	–0.54
Gender × smartphone experience	–0.23	0.08	–0.14	–2.87^∗∗^
Performance expectancy × age	–0.01	0.01	–0.12	−2.06^∗^
Effort expectancy × age	0.01	0.01	0.10	1.88
Social influence × age	0.01	0.01	0.12	1.95
Facilitating conditions × age	–0.01	0.01	–0.05	–0.76
Performance expectancy × gender	–0.13	0.11	–0.06	–1.15
Effort expectancy × gender	–0.29	0.16	–0.11	–1.80
Social influence × gender	0.07	0.12	0.03	0.61
Facilitating conditions × gender	0.42	0.16	0.16	2.65^∗∗^
Performance expectancy × smartphone experience	0.01	0.06	0.01	0.09
Effort expectancy × smartphone experience	–0.05	0.08	–0.04	–0.7
Social influence × smartphone experience	0.01	0.07	0.01	0.13
Facilitating conditions × smartphone experience	0.14	0.08	0.11	1.73
Age × gender × smartphone experience	0.01	0.01	0.06	1.09
Performance expectancy × age × gender	0.01	0.01	0.05	0.95
Effort expectancy × age × gender	0.03	0.01	0.17	2.46^∗^
Social influence × age × gender	–0.02	0.01	–0.11	−2.03^∗^
Facilitating conditions × age × gender	–0.03	0.01	–0.18	−2.60^∗^
Performance expectancy × age × smartphone experience	0.01	0.003	0.09	2.27^∗^
Effort expectancy × age × smartphone experience	–0.01	0.003	–0.09	–1.73
Social influence × age × smartphone experience	–0.004	0.004	–0.05	–1.04
Facilitating conditions × age × smartphone experience	0.004	0.004	0.05	1.00
Performance expectancy × gender × smartphone experience	–0.03	0.10	–0.014	–0.26
Effort expectancy × gender × smartphone experience	0.02	0.14	0.01	0.11
Social influence × gender × smartphone experience	0.09	0.12	0.04	0.79
Facilitating conditions × gender × smartphone experience	–0.22	0.15	–0.11	–1.46

**R*^2^ = 0.74, *F*(13, 358) = 2.171, *p* = 0.01. ^∗^*p* < 0.05, ^∗∗^*p* < 0.01, and ^∗∗∗^*p* < 0.001.*

In Step 1, we entered the main effects of age, gender, smartphone experience, and the four determinants. In Step 2, we added the two-way interactions between age, gender, and smartphone experience as well as between each of these variables and each determinant. In Step 3, we added the three-way interactions between age, gender, and smartphone experience as well as between pairs of these variables and each determinant. All variables were previously mean centered and the Process macro from SPSS was used to decompose significant interactions (Hayes, 2013). Because age is a continuous moderator, simple slopes were computed by selecting the conditional values of age as one standard deviation below the mean and one standard deviation above the mean (about 20 and 50 years, hereafter referred to as younger and older adults, respectively).

In Step 1, age, gender, smartphone experience, and ICT acceptance determinants explained 69% of the variance in behavioral intention to use mHealth apps, *R*^2^ = 0.69, *F*(7, 386) = 123.78, *p* < 0.001. Results showed significant main effects of age (*B* = 0.01, *p* < 0.001) and performance expectancy (*B* = 0.89, *p* < 0.001).

In Step 2, with the inclusion of the two-way interactions there was a significant increase of 3% in the explained variance, Δ*R*^2^ = 0.03, *F*_*change*_(15, 371) = 2.67, *p* = 0.001. Age (*B* = 0.02, *p* < 0.001) and performance expectancy (*B* = 0.90, *p* < 0.001) continued to contribute to the intention to use mHealth apps. Additionally, we found significant two-way interactions between age and three ICT acceptance determinants, namely, performance expectancy (*B* = −0.01, *p* = 0.02), effort expectancy (*B* = −0.01, *p* = 0.004), and facilitating conditions (*B* = −0.01, *p* = 0.009), as well as a significant interaction between facilitating conditions and gender (*B* = 0.35, *p* = 0.02).

**FIGURE 1:**
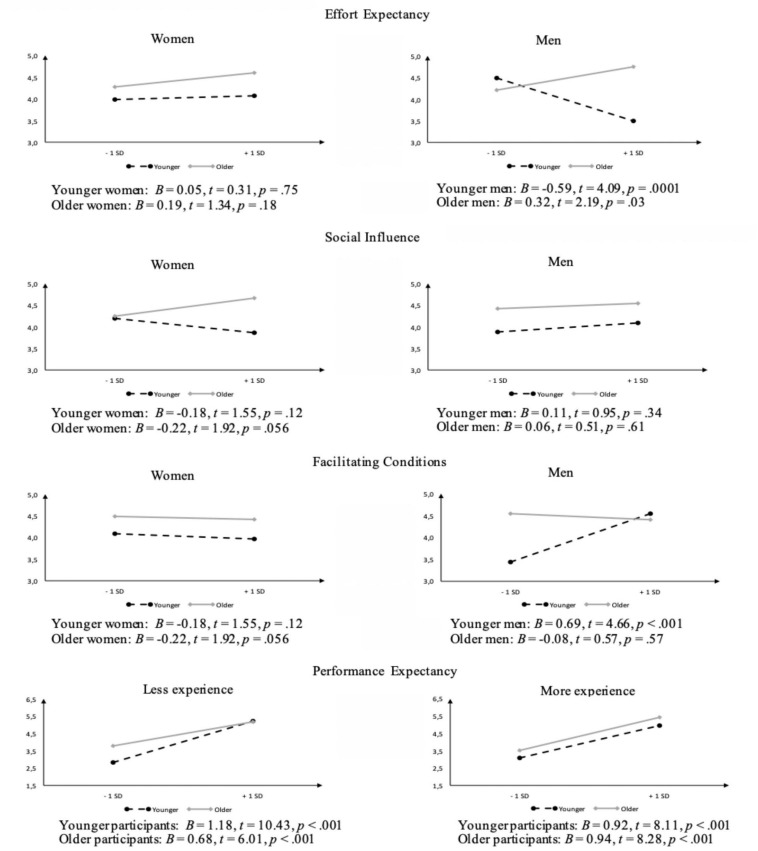
Graphs depicting the four significant three-way interactions.

In Step 3, including the three-way interactions resulted in a significant further increase of 2% in the explained variance, Δ*R*^2^ = 0.02, *F*_*change*_(13, 358) = 2.17, *p* = 0.01. The final model explained 74% of the variance. Age (*B* = 0.01, *p* < 0.001) and performance expectancy (*B* = 0.96, *p* < 0.001) remained significant predictors. Concerning the two-way interactions, we found a significant interaction between performance expectancy and age (*B* = −0.01, *p* = 0.04), as well as between facilitating conditions and gender (*B* = 0.42, *p* = 0.01). These two interactions were not decomposed because they were part of a three-way interaction (Aiken and West, 1991). There was also a significant interaction between gender and smartphone experience (*B* = −0.23, *p* = 0.004). Results decomposing this interaction showed marginally significant regression lines by gender. Smartphone experience was a positive predictor for women, *B* = 0.10, *t* = 1.87, *p* = 0.063, but a negative predictor for men, *B* = −0.10, *t* = 1.90, *p* = 0.0579. Finally, we found significant three-way interactions: effort expectancy × age × gender (*B* = 0.03, *p* = 0.01); social influence × age × gender (*B* = −0.02, *p* = 0.04); facilitating conditions × age × gender (*B* = −0.03, *p* = 0.01); and performance expectancy × age × smartphone experience (*B* = 0.01, *p* = 0.02). The results of these interactions are presented below (cf. Figure 1).

#### Effort Expectancy × Age × Gender

For women, effort expectancy did not contribute to the behavioral intention to use mHealth apps, neither in younger, *B* = 0.05, *t* = 0.31, *p* = 0.75, nor in older women, *B* = 0.19, *t* = 1.34, *p* = 0.18. For men, however, the effect of effort expectancy varied with age. In older men, effort expectancy was positively associated with the behavioral intention, *B* = 0.32, *t* = 2.19, *p* = 0.03, whereas in younger men it was the opposite – effort expectancy associated negatively with intention to use, *B* = −0.59, *t* = 4.09, *p* = 0.0001. Note that as effort expectancy is measured inversely, positive correlations mean that the easier the use is perceived, the stronger the intention.

#### Social Influence × Age × Gender

Social influence had no impact on the behavioral intention to use mHealth apps, neither in younger, *B* = 0.11, *t* = 0.95, *p* = 0.34, nor in older men, *B* = 0.06, *t* = 0.51, *p* = 0.61. It also had no impact in younger women, *B* = −0.18, *t* = 1.55, *p* = 0.12. However, in older women social influence was positively associated with the behavioral intention to use the apps, *B* = 0.22, *t* = 1.92, *p* = 0.056.

#### Facilitating Conditions × Age × Gender

For women, facilitating conditions did not contribute to the behavioral intention to use mHealth apps, neither in younger, *B* = −0.08, *t* = 0.51, *p* = 0.61, nor in older ones, *B* = −0.05, *t* = 0.33, *p* = 0.74. For men, however, the effect of facilitating conditions depended on age. In younger men, facilitating conditions associated positively with behavioral intention, *B* = 0.69, *t* = 4.66, *p* < 0.001, but they had no effect in older men, *B* = −0.08, *t* = 0.57, *p* = 0.57.

#### Performance Expectancy × Age × Smartphone Experience

Performance expectancy was positively associated with the behavioral intention to use mHealth apps in both younger and older participants regardless of smartphone experience. Stronger effects were found for younger participants with less smartphone experience, *B* = 1.18, *t* = 10.43, *p* < 0.001. We also found effects for participants with more smartphone experience, in both age groups: younger, *B* = 0.92, *t* = 8.11, *p* < 0.001, and older, *B* = 0.94, *t* = 8.28, *p* < 0.001. The smallest effect was observed in older participants with less smartphone experience, *B* = 0.68, *t* = 6.01, *p* < 0.001.

## Discussion

Our goal was to examine the moderating roles of age, gender, and smartphone experience in the relationship between the four ICT acceptance determinants from UTAUT and the behavioral intention to use mHealth apps. Overall, the model including only main effects, and the final model including also moderating effects, explained 69 and 74%, respectively, of the variance in the behavioral intention to use mHealth apps. This large amount of explained variance is in agreement with the figure found for the original UTAUT model applied to organization settings (Venkatesh et al., 2003). Additionally, our moderation analyses revealed that the effects of the four determinants on the intention to use mHealth apps were all in the form of three-way interactions.

### Performance Expectancy

Performance expectancy was the only determinant to exert a main effect on the behavioral intention to use mHealth apps. This result is consistent with findings from studies on mHealth apps (Yuan et al., 2015) and other types of ICT, such as home telehealth services (Cimperman et al., 2016), or mobile internet (Wang and Wang, 2010). This effect was moderated by age and smartphone experience (not by gender). Performance expectancy significantly contributed to the behavioral intention to use mHealth apps in both younger and older adults regardless of smartphone experience. An examination of effect sizes revealed that with less experience the effect was stronger for younger than older adults (*B* = 1.18 vs. 0.68). Note that few studies have assessed the role of ICT experience in technology acceptance (Williams et al., 2015). An exception is Al Awadhi and Morris’s (2008) study on the behavioral intention to use e-government services, where internet experience was a significant moderator of performance expectancy.

### Effort Expectancy

The effect of effort expectancy on the intention to use mHealth apps was moderated by age and gender (not by experience). Effort expectancy had a significant contribution only among men, and this contribution depended on their age. In line with Venkatesh et al. (2003) and Cimperman et al. (2016), here too effort expectancy was particularly relevant for older men. The more older men perceived mHealth apps as easy to use, the more they intended to use it. The opposite happened with younger men: the more they perceived the ICT as easy, the less they intended to use it. To the best of our knowledge, this is the first time that such a negative effect is reported. A possible explanation is that younger men might have interpreted ICT ease of use with simple-mindedness or lack of sophistication of the technology. For example, the item “I find that using mHealth apps would be simple” might have been understood as meaning an app with narrow functionalities. In that case, by considering mHealth apps as less challenging and interesting than other types of ICT, younger men might have been less motivated to adhere to them. Indeed, hedonic motivation, defined as the fun or pleasure derived from using a technology, may underlie the decision to use ICT (Venkatesh et al., 2012). It has been suggested that, besides looking at utilitarian benefits and ease of use, younger men tend to seek ICT innovativeness when contemplating using new apps (Venkatesh et al., 2012).

### Social Influence

We found stronger effects of social influence on the intention to use mHealth apps among older women. The significant and positive effect of social influence on women’s intention is consistent with other studies (Wills et al., 2008; Sun et al., 2013). It seems that women’s sensitivity to others’ opinions leads them to value more those opinions when making decisions about adopting ICT (Venkatesh and Davis, 2000). Highlighting the importance of taking into account user characteristics when considering ICT acceptance, we found no main effect of social influence on the behavioral intention to use mHealth apps. This fits well with previous findings of no effect of social influence on the intention to use ICT apps, not only in organization settings where use is often mandatory, but also in settings where use is voluntary (Arenas-Gaitán et al., 2015).

### Facilitating Conditions

Our findings also showed that the influence of facilitating conditions was moderated by age and gender. It was stronger for younger men: perceiving availability of resources and support is a relevant condition for younger men to be interested in using mHealth apps. However, it should be noted that research into the role of facilitating conditions has typically focused on actual use of ICT rather than on behavioral intentions (Williams et al., 2015). Among the few studies focusing on these, the moderating role of user characteristics has barely been tested. Clearly, more attention is needed on how perceived technical support shapes users’ intentions to use ICT.

### Age Effects on the Behavioral Intention to Use mHealth Apps

Despite the importance given to age in the UTAUT model, this variable has received little attention (Lee et al., 2003; Venkatesh et al., 2016). In our study, age was a significant moderator in the relationship between the four ICT-acceptance determinants and the behavioral intention to use mHealth apps. Thus, age should be taken into account when considering ICT acceptance, and its role might be particularly important if the target population is gender specific. Levy (1988) has suggested that when testing for gender differences, leaving age out of the equation may be misleading. In our study, age and gender together moderated the effect of three determinants: effort expectancy, social influence and facilitating conditions. The behavioral intention to use ICT was influenced by effort expectancy in older men (less effort, more use) and younger men (negatively), by social influence in older women (positively), and by facilitating conditions in younger men (positively).

### Limitations and Future Directions

The previously discussed findings should be considered in view of four limitations. First, as data were obtained at a single point in time and the study was correlational, causality inferences are not warranted. Further research is needed to replicate these findings and to tap the causal mechanisms through which ICT acceptance determinants affect the behavioral intention to use mHealth apps. Second, we did not measure effective use of mHealth apps; instead, we focused on people who were not currently using mHealth apps and on their intentions to do so in the future. Further research should include users of mHealth apps and possibly compare them with non-users. Third, due to the recruitment procedure our sample included more women than men. Future studies should aim to collect larger samples with an equivalent number of men and women. Finally, our variable experience was restricted to smartphone use and was not measured longitudinally as proposed in the UTAUT model.

## Conclusion

Our findings relate to the moderating role of age, gender, and experience on the intention to use mHealth apps. The focus on the study of personal characteristics is important to develop mHealth apps adjusted to potential users. Our findings showed that the UTAUT model is a useful tool to test the behavioral intention to use mHealth apps, and provided additional support to extant literature on the role of age, gender, and experience as significant moderators. This knowledge is an asset for the development of new health-related ICT.

## Author’s Note

A subgroup of this sample was used in a preliminary study presented at the “4th International Conference on Information and Communication Technologies for Ageing Well and e-Health” and published as “Nunes, A., Limpo, T., & Castro, S. L. (2018). Effects of age, gender, and personality on individuals’ behavioral intention to use health applications. In *Proceedings of the 4th International Conference on Information and Communication Technologies for Ageing Well and e-Health (ICT4AWE 2018)*, SCITEPRESS – Science and Technology Publications, pages 103–110.” Using a more sophisticated approach, the results from this preliminary study were subsequently published in a book chapter as “Nunes A., Limpo T., Castro S. L. (2019). Individual factors that influence the acceptance of mobile health apps: The role of age, gender, and personality traits. In P. D. Bamidis, M. Ziefle, & L. Maciaszek (Eds), *Information and Communication Technologies for Ageing Well and e-Health* (pp. 167–179). Cham: Springer. Doi:10.1007/978-3-030-15736-4_9” These two prior studies compared younger and older adults (18–39 years vs. 40–65 years) on the behavioral intention to use mHealth apps.

## Data Availability Statement

The datasets generated for this study are available on request to the corresponding author.

## Ethics Statement

The studies involving human participants were reviewed and approved by the Comissão de Ética para a Saúde do Centro Hospitalar S. João (CES). The patients/participants provided their written informed consent to participate in this study.

## Author Contributions

AN, TL, and SC designed the study and translated the questionnaire independently. AN collected and coded the data of the study. AN and TL analyzed and interpreted the data. AN drafted the manuscript. TL and SC edited and reviewed the manuscript. All authors read and approved the final version of the manuscript.

## Conflict of Interest

The authors declare that the research was conducted in the absence of any commercial or financial relationships that could be construed as a potential conflict of interest.

## Footnotes

1 Research2Guidance is a strategy advisory and market research company, focused in the mobile app eco-system. More information can be found here: https://research2guidance.com/product/mhealth-economics-2017-current-status-and-future-trends-in-mobile-health/.

2 Voluntariness is the mandatory or voluntary nature of ICT use. This distinction makes sense in organizations, where the UTAUT model was originally developed. However, in the case of mHealth apps the use of ICT is voluntary and so this moderator makes no sense. Thus, voluntariness was not considered in the present study.
