# Supplementary material for: Acceptance of Mobile Health Applications: Examining Key Determinants and Moderators
Source: Front Psychol. 2019 Dec 10;10:2791. doi: 10.3389/fpsyg.2019.02791 (PMC6914844; doi:10.3389/fpsyg.2019.02791)
Supplement: Supplementary file 1 [file Table_1.DOCX]

Supplementary Material

Acceptance of mobile health applications: Examining key determinants and moderators

Andreia Nunes, Teresa Limpo, São Luís Castro^*^

*** Correspondence:** São Luís Castro: slcastro@fpce.up.pt

# Supplementary Table

Table S1: Technology Acceptance Factors, Original English Items and Corresponding Translation to Portuguese. Note. ^1^HTS refers to Home Telehealth Services (HTS), the technology studied by Cimperman et al. (2016). We rephrased the items to focus on mHealth applications, translated into Portuguese as “aplicações de saúde”. Rated from “strongly disagree” (1) to “strongly agree” (7). The following definition of mHealth applications was provided to participants: “mHealth apps are intended to promote the health of the users, allowing them to record and monitor aspects of their health, such as sleep (e.g., number of hours slept), physical activity (e.g., number of steps), food (e.g., calories ingested), vital signs (e.g., blood pressure), or reproductive health (e.g., menstrual cycle).”

| Factors | Original English Items (Cimperman et al., 2016) | Portuguese items for mHealth apps |
| --- | --- | --- |
| Performance Expectancy | HTS^1^ could enhance the quality of my life. | 1. As aplicações de saúde poderiam melhorar a minha qualidade de vida. |
|  | I find that using HTS would be helpful in monitoring my health. | 6. Penso que usar aplicações de saúde seria útil para saber como anda a minha saúde. |
|  | I find that using HTS would make me feel safer in my daily life. | 11. Penso que usar aplicações de saúde me iria fazer sentir mais seguro no meu dia-a-dia. |
|  | HTS could enhance the level of convenience in accessing medical care services. | 16. As aplicações de saúde poderiam facilitar o meu acesso aos serviços de cuidados médicos. |
|  | Overall, I find HTS would be highly useful. | 18. Em geral, penso que as aplicações de saúde poderiam ser muito úteis. |
| Effort Expectancy | I find that HTS would be easily understandable and clear for me. | 3. Penso que teria facilidade em perceber para que servem as aplicações de saúde. |
|  | Overall, I find that using HTS would be convenient. | 8. Em geral, penso que seria vantajoso usar aplicações de saúde. |
|  | I find that using HTS would be simple. | 13. Penso que seria simples usar aplicações de saúde. |
|  | I find that using HTS would be easy to learn. | 17. Penso que seria fácil aprender a usar aplicações de saúde. |
| Social Influence | Peers and colleagues would support me in using HTS. | 2. As pessoas da minha idade iriam apoiar a minha decisão de usar aplicações de saúde. |
|  | People who influence my behavior would support me using HTS. | 7. As pessoas que influenciam o meu comportamento iriam apoiar a minha decisão de usar aplicações de saúde. |
|  | People who are important to me would support my use of HTS. | 12. As pessoas que são importantes para mim iriam apoiar a minha decisão de usar aplicações de saúde. |
| Facilitating Conditions | I believe guidance will be available to me when deciding whether to use the system. | 4. Acredito que terei ajuda se decidir usar aplicações de saúde. |
|  | I believe specific persons (or a group) will be available for assistance with system difficulties (a call center). | 9. Acredito que haverá pessoas disponíveis para me ajudar se tiver dificuldades ao usar aplicações de saúde (ex.: call center, familiar). |
|  | I believe specialized instructions concerning use of the system will be available to me. | 14. Acredito que haverá instruções detalhadas sobre como usar as aplicações de saúde. |
| Behavioral Intention | Assuming I had access to an HTS system, I would intend to use it. | 5. Se tiver acesso a aplicações de saúde tenho intenção de utilizá-las. |
|  | I predict I will use an HTS system on a regular basis in the future. | 10. Penso que no futuro irei utilizar aplicações de saúde regularmente. |
|  | I intend to use an HTS system in the future. | 15. Tenciono usar aplicações de saúde no futuro. |
|  | Given that I had access to an HTS system, I would use the services. | 19. Supondo que terei acesso a aplicações de saúde, iria utilizar estas aplicações. |
